# An Overview of Oral Pyogenic Granuloma and Its Management: A Case Report

**DOI:** 10.7759/cureus.48305

**Published:** 2023-11-05

**Authors:** Mrunal Meshram, Khushboo Durge, Unnati Shirbhate

**Affiliations:** 1 Department of Oral Medicine and Radiology, Sharad Pawar Dental College, Datta Meghe Institute of Higher Education and Research, Wardha, IND; 2 Department of Periodontics, Sharad Pawar Dental College, Datta Meghe Institute of Higher Education and Research, Wardha, IND

**Keywords:** periodontal surgery, pregnancy tumor, hyperplastic lesion, oral pyogenic granuloma, pyogenic granuloma

## Abstract

An inflammatory hyperplasia known as a pyogenic granuloma (PG) appears as a nodular growth on the oral mucosa. The most frequent place is the gingiva, followed by the buccal mucosa, tongue, and lips. Histologically, the surface epithelium may be hyperkeratotic, have ulceration foci, or be intact. It lies on the dense connective tissue that contains a sizable amount of fully developed collagen. Most of the pregnancies result in PG of the gingiva; for this reason, the phrases "Pregnancy Tumor" and "Granuloma Gravidarum" are frequently used. It typically occurs during the second and third months of pregnancy and tends to bleed, making it challenging to masticate. Estrogen increases the vascular endothelial growth factor (VEGF) synthesis in macrophages, which is associated with PG development during pregnancy. This case describes a surgically treated case of PG in a middle-aged female with a conventional scalpel technique, giving functional and esthetic outcomes in a patient.

## Introduction

Pyogenic granuloma (PG) frequently encounters non-neoplastic tumor-like growth of the oral tissues [1,2]. Oral PG commonly arises in response to various stimuli, likely low-medium grade local irritation, any traumatic injuries, hormone-related factors, or may be due to some kinds of medications [3-5]. The main etiologic factor was thought to be caused by pyogenic organisms, but now it is believed to be unrelated to infection. Therefore, the term "pyogenic granuloma" became a misnomer as it neither contains the pus nor is strictly a granuloma histologically [2,6]. PG is known to involve the gingiva mainly [7]. Apart from gingiva, it can also affect the lips, tongue, buccal mucosa, and palate. PGs are generally present as soft, painless, pedunculated, or sessile gingival masses and deep red to reddish-purple [8,9]. The current case report depicts clinical and radiological features, histological characteristics, and a suitable treatment modality for a typical PG lesion in a middle-aged female patient.

Oral PGs are the most common hyperplasic reactive lesions in the mouth and are considered non-neoplastic [10]. Bhaskar et al. reported that PG accounts for 1.85% of all oral pathologies [11]. Irritation factors can be dental calculi, over-contoured restorations, gingival trauma due to deciduous teeth, failure of teeth eruption, dental implants foreign bodies, poor oral hygiene, some unspecified infection, immunosuppression, and in a few cases, hormonal disturbances [12]. PG is also known as "granuloma gravidarum, eruptive hemangioma, granulation tissue hemangioma, lobular capillary hemangioma, or tumor of pregnancy". It can occur at any age, but more documented cases have been seen in the second-third decades of life [13]. Females have a slight predilection over males [14]. It is mainly observed in the first trimester of pregnancy. The possible cause is stimulating the expression of angiogenic factors in inflammatory tissues by elevated levels of estrogen and progesterone, leading to vascular morphogenesis [14]. Radiographic findings of PG are generally absent except for localized alveolar bone resorption in long-standing lesions [15]. The clinical presentation shows an elevated, smooth exophytic, sessile or pedunculated mass of growth covered with reddish hemorrhagic and compressible papules, which appear lobulated and may be covered by a yellow fibrinous membrane [3]. Depending on the degree of vascularity of the growth, the color varies from reddish purple to pink. The gingiva, especially the marginal gingiva, is affected more than the alveolar part, and its size varies from a few millimeters to several centimeters [3]. This case describes the clinical features and histopathological features showing PG, which was treated with a conventional scalpel approach with complete satisfactory healing as well as no discomfort, pain, scar, and no recurrence signs on excision of the lesion.

## Case presentation

**Figure 1:**
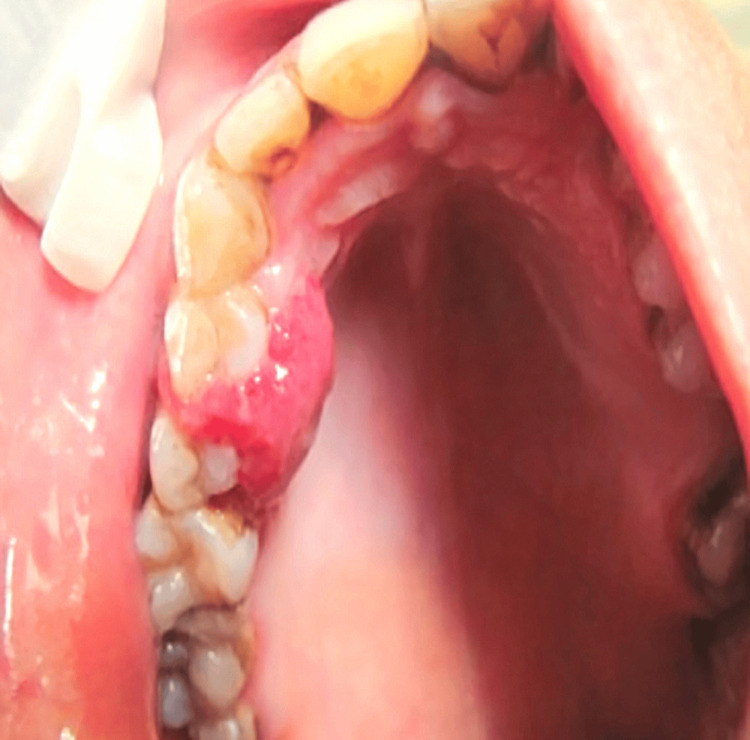
Pre-operative view of pyogenic granuloma

The primary complaint of a 40-year-old female patient who visited the Department of Oral Medicine was a painful gum swelling in the upper right jaw area that had been bothering her while eating for two to three months. It started out being small and grew more prominent over time. She had visited a doctor one month before reporting to our Outpatient Department, who had given her multivitamin capsules and gum paint for topical application. She received no solace. She had stopped brushing that area since it was bleeding. The patient did not give any history of systemic illness. On extra-oral examination, no swelling on the right-sided maxilla was seen. A solitary right submandibular lymph node was palpable at approximately 0.5x0.5 cm, roughly oval, soft, mobile, and non-tender. Intraoral examination in Figure 1 revealed well-defined sessile growth in the right maxillary canine-premolar-molar region extending from the palatal and buccal region, involving marginal and attached gingiva. It was reddish pink in color, ovoid in shape, and approximately 2.5x3 cm in size. The surface was smooth, and no ulcerations were seen. 

**Figure 2:**
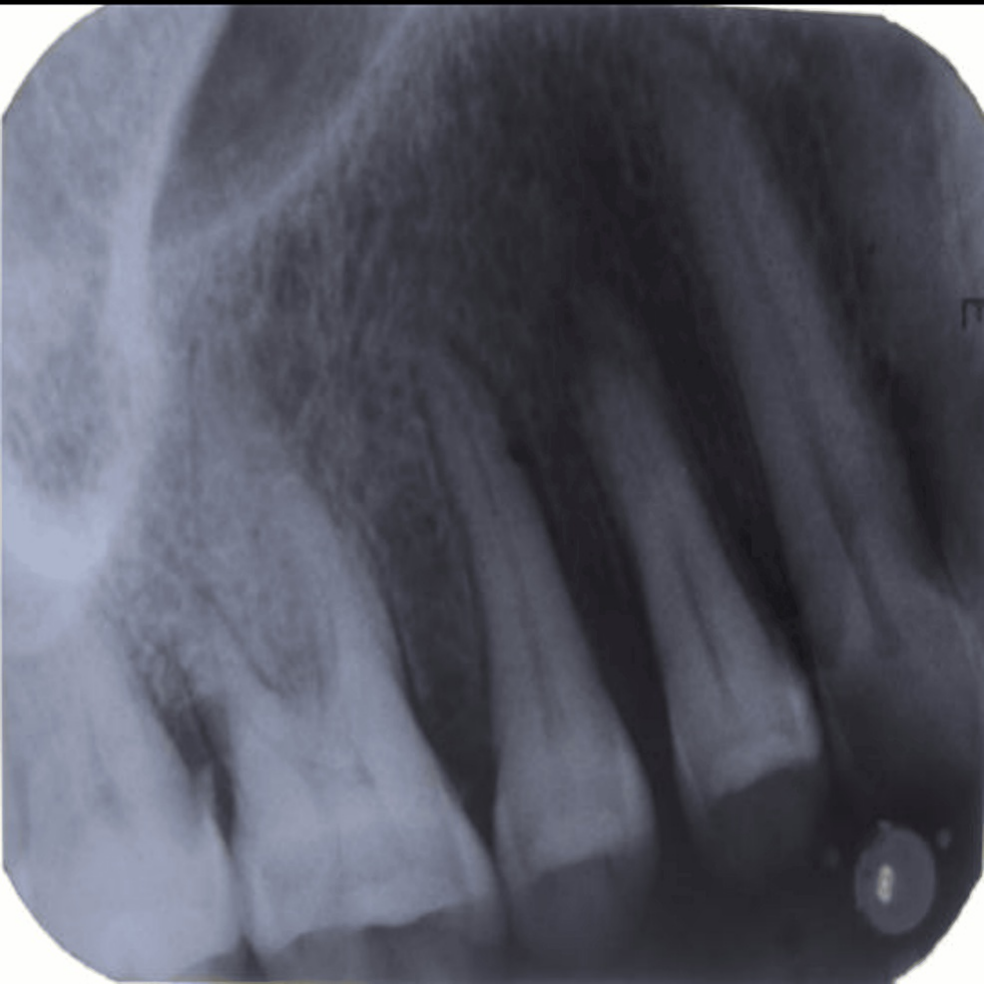
Radiographic changes of the pyogenic granuloma with respect to the affected area

It was tender on palpation with a soft consistency. Poor oral hygiene and calculus were present. The teeth around the lesion showed no signs of mobility. Except for periodontal widening with roots of 15 and 16 and mild alveolar bone loss in the growth zone, there were no apparent abnormalities on radiographs, as shown in Figure 2. A typical hemogram was discovered to be anticipated. The diagnosis of PG was made. Peripheral giant cell granuloma and peripheral ossifying fibroma were included in the differential diagnosis. Periodontal therapy has always aimed to reduce bacterial infection by the mechanical removal of infectious pathogens. Therefore, a complete oral prophylaxis protocol was followed, and the case was prepared for surgery based on the clinical and radiographic evidence. After the completion of phase I therapy, the follow-up was taken. Thus, the periodontal examination revealed an excellent plaque index score of <1 by Silness and Loe in 1964 and mild gingival inflammation as per a gingival index score of <1 by Loe and Silness in 1963. Therefore, it was decided to proceed with the surgical phase.

**Figure 3:**
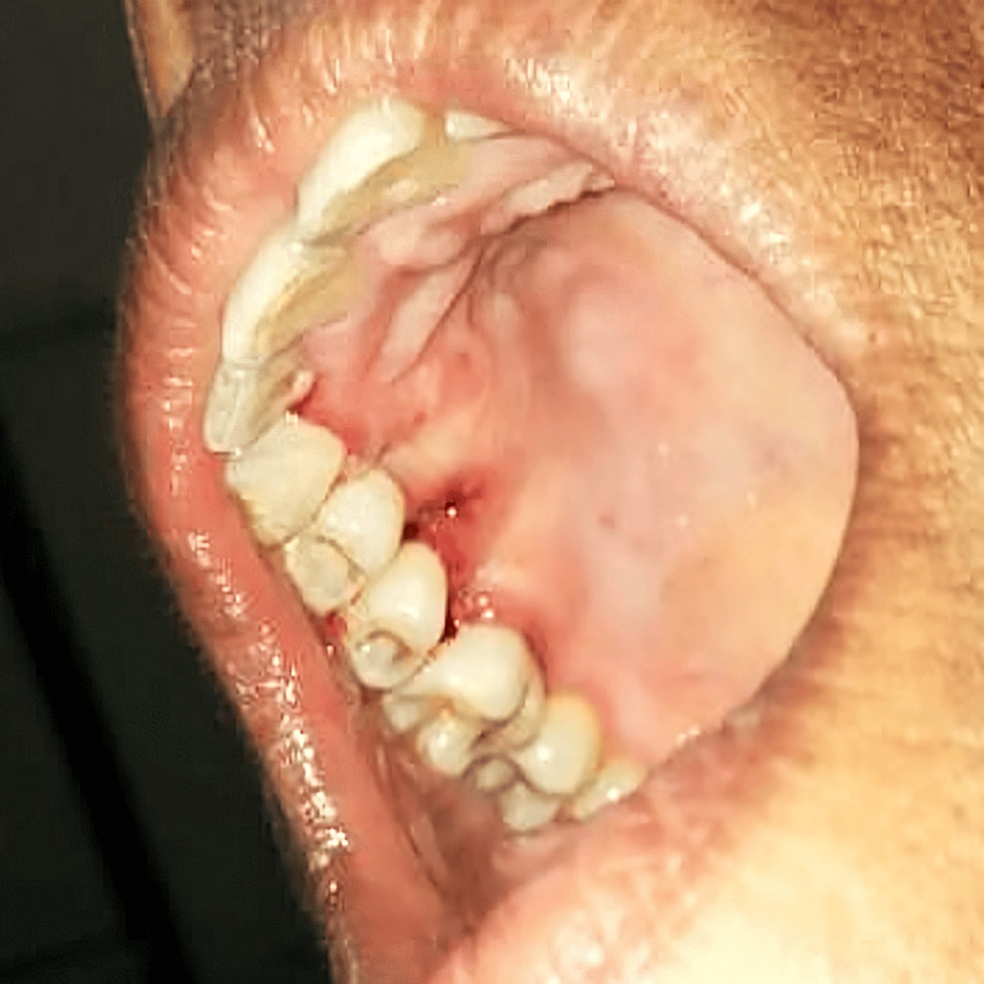
Surgical excision is done by the scalpel of the pyogenic granuloma

A conventional surgical procedure by scalpel method was chosen for the excision of the lesion. The blood investigations were performed to rule out the range of hemoglobin, bleeding time, and clotting time. The results of the blood investigations were within normal limits. A written, signed informed consent was taken from the patient before the surgical procedure. An area was anesthetized with a solution of 2% lignocaine with 1:200000 adrenaline under all aseptic precautions. The lesion was then demarcated. A circumferential full-thickness incision was given. The incision extended from the distal aspect of the canine (maxillary right) to the mesial part of the first molar (maxillary right) region. Scalpel excision was performed from the base of the lesion. After excision, open flap debridement was done to exclude diseased granulation tissue and scrape out diseased periosteum in the surrounding area adjacent to the lesion. Figure 3 shows the postoperative view of PG.

**Figure 4:**
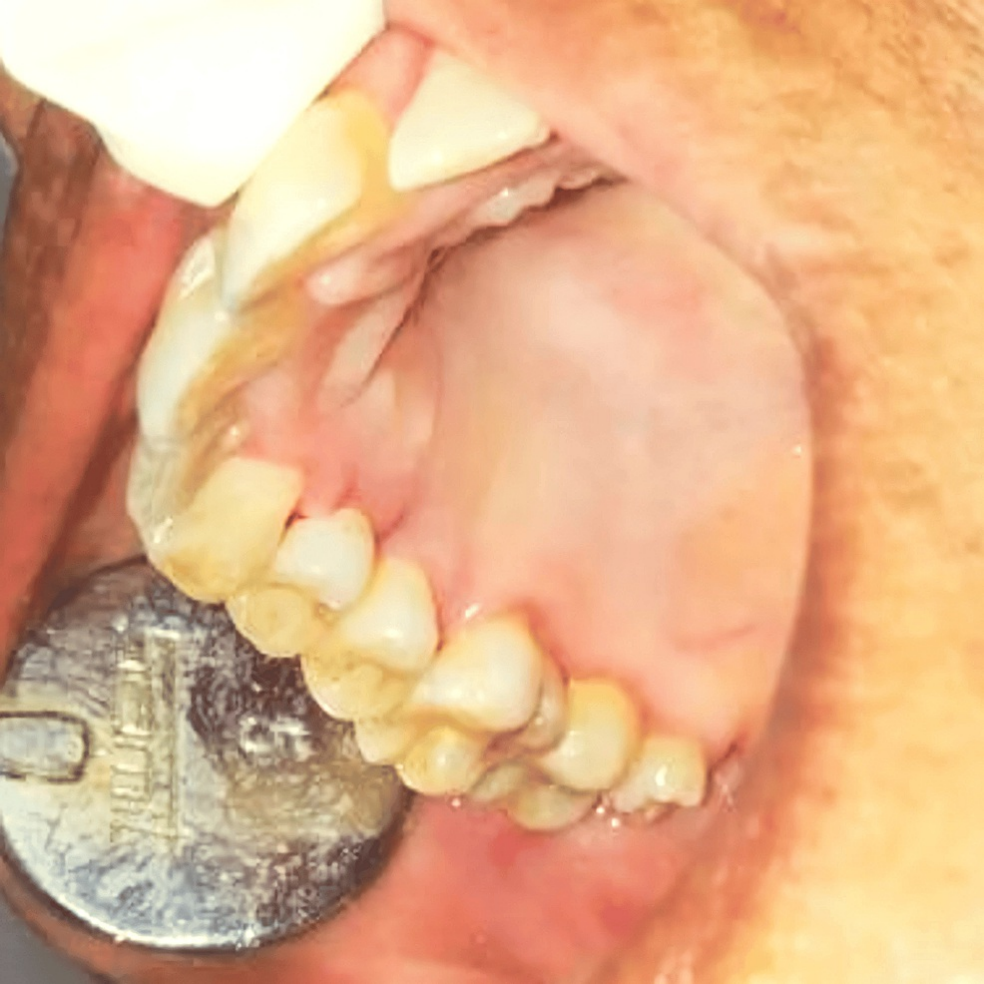
Recall examination after seven days revealed complete healing of the pyogenic granuloma

The excised specimen was then transferred to a bottle of 10% formalin solution and was sent for histopathological evaluation. After thorough curettage was performed, the available healthy soft tissue was sufficiently covering the operated site; the flap was then coronally approximated by placing simple interrupted three to zero silk sutures. The periodontal dressing was placed. Postoperative instruction was given to the patient. An antibiotic and analgesic coverage (amoxicillin 500 mg thrice daily for five days and ibuprofen 400 mg thrice daily for five days) and chlorhexidine mouthwash (0.12%) were prescribed to the patient for five days. Sutures were removed after a week. On the seventh day, the patient showed proper region healing with improved oral hygiene status, as shown in Figure 4.

**Figure 5:**
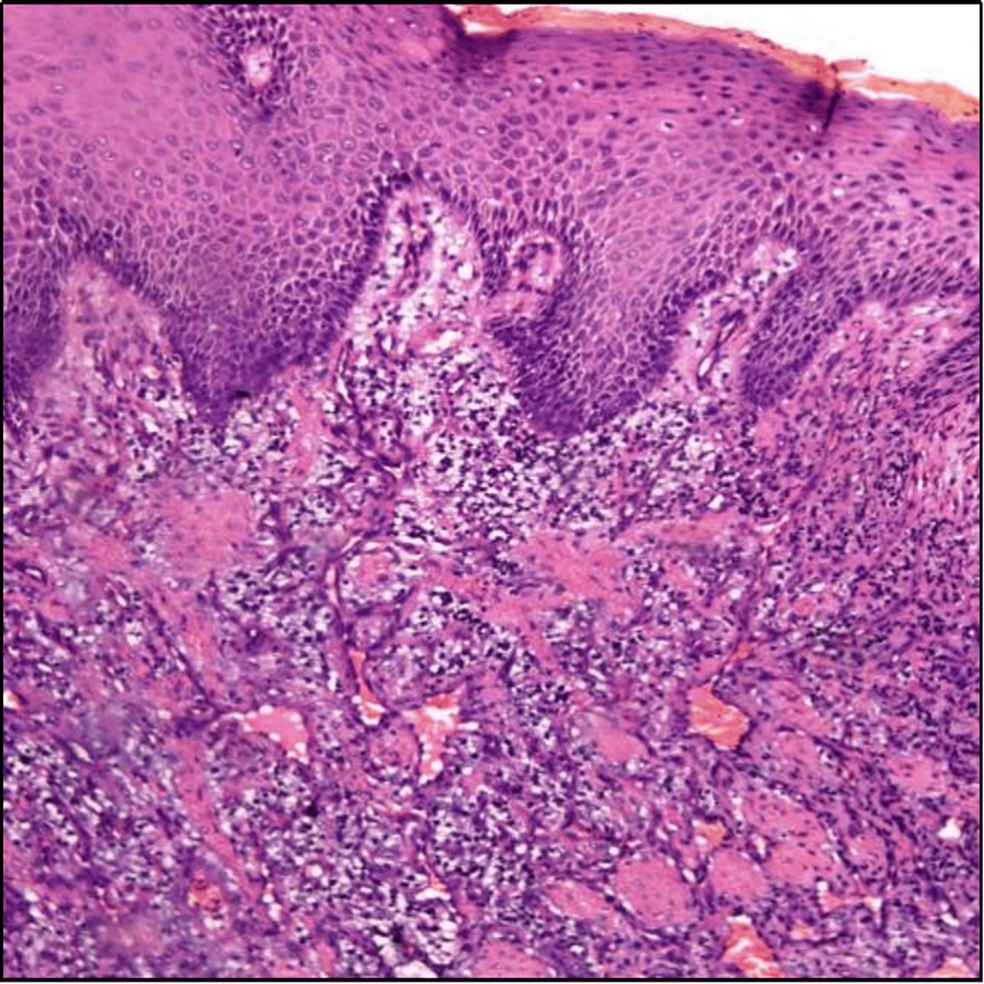
Histologic findings of pyogenic granuloma

Histopathological criteria are needed to distinguish similar features. Thus, histopathological investigations were carried out. Histopathological evaluation revealed a single bit of tissue section overlying epithelium and underlying connective tissue stroma. Overlying para-keratinized stratified squamous epithelium was of variable thickness at places. The surrounding connective tissue stroma showed intense chronic inflammatory cell infiltration, predominantly of lymphocytes. Also, numerous thin dilated blood vessels with intravasated and extravasated red blood cells were seen. The connective tissue stroma appeared myxoid in places. All these findings of low power view were confirmed in high power, and the features were suggestive of PG, as appreciated in Figure 5. So, based on a histological report, the final diagnosis was given of PG.

## Discussion

The case presented here showed solitary, reddish pink, oval, sessile growth, approximately 2.5x3 cm, involving marginal and attached gingival, which was tender with a soft consistency. Histological variants of it are lobular and non-lobular. A more significant number of proliferating blood vessels with mild or no specific changes suggests lobular form. At the same time, the presence of dilated capillary channels and alignment with the endothelial cells indicates a non-lobular form [16]. About the differential diagnosis, mention should be made for “peripheral giant cell granuloma, peripheral ossifying fibroma, peripheral fibroma, Kaposi's sarcoma, angiosarcoma, metastatic carcinoma, non-Hodgkin's lymphoma, and hemangioma” [17]. The diagnosis of PG can often be challenging because of the similar clinical presentation of many other intraoral angiomatous lesions. Thus, there is a need for in-depth clinical examination. Clinical and histological features are used for differentiation, which aids in treatment planning and, consequently, improves prognosis. PG treatment entails removing all etiological causes and performing a total surgical excision to lower the chance of recurrence. Traditionally, incisional procedures are made with a stainless-steel scalpel [18]. Asnaashari et al., 2014, reported that the lesion recurrence rate following the conventional method was 16%. Since the lesion in our patient had a sessile basis and a history of recurrent recurrence, the standard scalpel method was recommended. To stop the lesion from returning, the entire underlying soft tissue was removed during the excision [19]. There was no recurrence noted during the maintenance session. Most of the PGs appearing during pregnancy subside after delivery. If the lesion is large or associated with bleeding, treatment during pregnancy is recommended in the second trimester, with ongoing checks after delivery. Also, diode or CO_2_, cryosurgery, flashlamp-pumped pulsed dye laser, laser resection, intra-lesional injection of corticosteroids or sclerosing agents, and nitrogen cryosurgery are the treatments of choice [14,17].

## Conclusions

This case discusses the lesion's clinical and histological characteristics and notes that, despite being often used, the name "pyogenic granuloma" is incorrect because it is not pus-associated and is histologically angiomatous rather than granulomatous. Despite new treatment options, surgical excision will continue to be the treatment of choice because it can be completed in a single session using standard surgical instruments instead of other procedures that call for multiple appointments, specialized staff training, and expensive equipment. Also, it is easily applied in routine clinical practice, and recurrence is rare after surgical excision. This case demonstrates complete satisfactory healing after the excision of pyogenic granuloma by the conventional scalpel method with no scar tissue formation, no discomfort, no pain, and relieving the symptoms related to mastication, giving esthetic and functional demands of the patient. 
